# Effects of Intermittent Fasting‐Mimicking Diet on Pancreatic Islet Plasticity: Immunohistochemical, Ultrastructural, and Metabolic Profiles

**DOI:** 10.1096/fj.202504830RR

**Published:** 2026-05-04

**Authors:** Clemens M. Harer, Beate Boulgaropoulos, Barbara Ehall, Lea Bogensperger, Kaddour Bounab, Dominique Pernitsch, Joakim Franz, Laurin Herbsthofer, Petra Kotzbeck, Jelena Krstic, Andreas Prokesch, Barbara Prietl, Dagmar Kolb, Thomas R. Pieber

**Affiliations:** ^1^ Division of Endocrinology and Diabetology Medical University of Graz Graz Austria; ^2^ HEALTH‐Institute for Biomedical Research and Technologies, Joanneum Research Forschungsgesellschaft mbH Graz Austria; ^3^ BioTechMed Graz Austria; ^4^ TU Graz, Institute of Computer Graphics and Vision Graz Austria; ^5^ Gottfried Schatz Research Center for Cell Signaling, Metabolism, and Aging, Division of Cell Biology, Histology, and Embryology Medical University of Graz Graz Austria; ^6^ Center for Biomarker Research in Medicine GmbH Graz Austria; ^7^ Division of Plastic, Aesthetic and Reconstructive Surgery Medical University of Graz Graz Austria

**Keywords:** β‐cell, diabetes, fasting‐mimicking diet, FMD, insulin, pancreas, pancreatic islet plasticity, prolonged periodic intermittent fasting, refeeding

## Abstract

Periodic fasting is known to improve metabolic health, but its impact on pancreatic islet plasticity remains unclear. We investigated the effects of intermittent fasting‐mimicking diet (FMD) cycles on islet architecture and function in mice by performing immunohistochemical, ultrastructural, and metabolic analyses after fasting and after refeeding separately. Twelve‐week‐old female C57BL/6J mice were randomized to fasting (*n* = 9), refeeding (*n* = 10), or control group (*n* = 10). FMD was supplied weekly for 3 days (50%, 10%, 10% of daily caloric intake) followed by 4 days of chow food ad libitum (except for pre‐IGTT food withdrawal and the duration of the IGTT). Intraperitoneal glucose tolerance tests (IGTTs) were performed at day 11 (fasting group), day 14 (refeeding group), and day 13 (control group). Mice were sacrificed 7 days after IGTT, and pancreata were subjected to fluorescence immunohistochemistry or scanning electron microscopy (STEM). Bodyweight, blood glucose, proinsulin, and IGF‐1 concentrations were significantly decreased after fasting but rebounded after refeeding. Pancreatic insulin^+^glucagon^+^, BRN4^+^, and PDX1^+^BRN4^+^ cells increased significantly after fasting and tended to remain high after refeeding, thereby indicating increased pancreatic islet plasticity after fasting. In STEM images, the insulin granule core‐to‐halo ratio increased significantly after fasting. The fasting but not the refeeding group showed impaired glucose tolerance. The more crystallized mature β‐cell granules indicate increased insulin secretory capacity, and the reduced proinsulin‐to‐insulin ratio suggests reduced endoplasmic reticulum stress in ß‐cells after fasting. We propose that this observed plasticity may provide a basis for novel concepts of in vivo β‐cell regeneration. However, further studies to investigate molecular mechanisms of fasting/refeeding in murine type 1 diabetes to evaluate its therapeutic potential are needed.

## Graphical abstract

Three weeks of fasting‐mimicking diet in female C57BL/6J mice increased pancreatic BNR4^+^ cells, PDX1^+^BRN4^+^ cells and insulin^+^glucagon^+^ cells after fasting in fluorescent multiplexed immunohistochemistry. Mice showed impaired glucose tolerance after fasting but not after refeeding after 2 weeks. Increased core‐to‐halo ratio in scanning electron microscopy indicated more crystallized mature β‐cell granules and reduced ER‐stress in ß‐cells after fasting.

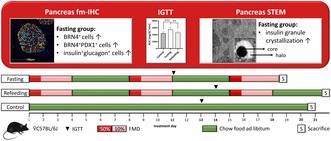

## 1 Introduction

Energy storage and metabolic adaptations to periods of food deprivation and abundance have been the key to evolution and survival of mammals [1]. Nowadays, however, people in Western countries have constant and easy access to high‐caloric food which minimizes periods of food deprivation [2, 3], and which, in combination with a sedentary lifestyle, contributes to a high prevalence of metabolic diseases such as obesity and diabetes mellitus [4, 5].

These detrimental effects can be counteracted with dietary interventions such as intermittent fasting or caloric restriction, both of which have been associated with a wide range of health benefits [6, 7, 8] such as improvements in Alzheimer's disease [9], improved immune function [10, 11, 12], as well as improved insulin resistance and Type 2 diabetes (T2D) [13]. Although intermittent fasting has also reduced the daily insulin requirements in people with Type 1 diabetes (T1D) and does not appear to lead to diabetic ketoacidosis under clinically controlled conditions [14], it remains unknown whether fasting and refeeding can induce the formation of new functional β‐cells in humans. However, because β‐cells are highly sensitive to the availability of glucose, the implementation of fasting and refeeding as a prospective therapeutic approach in T1D and T2D could result in reprogramming of pancreatic progenitor cell lines and possibly in the regeneration of pancreatic β‐cells [15].

Nevertheless, the positive effects of fasting on health and disease often cannot fully unfold because adherence to prolonged fasting regimens is challenging for most individuals [16]. To ease the hardship of prolonged fasting and reduce the risk of malnourishment during water‐only fasting, a commercially available diet has been developed that reproduces key physiological effects of fasting (fasting‐mimicking diet, FMD) [17]. When FMD is administered during fasting cycles of 3 to 4 days followed by refeeding, mice have been shown to have extended longevity, reduced visceral fat and reduced cancer incidence [17]. In humans, FMD treatment applied during fasting/refeeding cycles has exhibited beneficial effects in the treatment of breast cancer [18, 19] and relapsing‐remitting multiple sclerosis [20], and has led to reduced insulin resistance and hepatic lipid content [21]. A most recent study has demonstrated that short‐term FMD treatment increases the expression of genes driving gluconeogenesis and fatty acid oxidation in mice [22].

In T1D and T2D mouse models, prolonged FMD treatment during three fasting/refeeding cycles has been shown to promote the expression of the transcription factor Neurogenin3 (Ngn3) in the endocrine pancreas, thereby increasing insulin production and reversing hyperglycemia [23]. Since the effects of FMD treatment have been partially reversed under insulin‐like growth factor 1 (IGF‐1) treatment in that study, it has been hypothesized that energy restriction and low protein contents of the diet can lead to decreased IGF‐1 concentration during fasting. Lower IGF‐1 concentrations may, in turn, suppress β‐cell senescence and improve β‐cell function [23, 24, 25]. Further evidence for the expression of Ngn3 in the endocrine pancreas and distinct changes in gut microbiota of T2D mouse models have been demonstrated after FMD treatment [26]. However, despite these observed beneficial effects of fasting/refeeding on β‐cell regeneration, we suggest that pancreatic β‐cells might exhibit substantially more plasticity than is generally assumed.

The aim of this study was therefore to gain a deeper understanding of pancreatic islet plasticity by assessing immunohistochemical, ultrastructural, and metabolic profiles after and during three intermittent FMD cycles, employing a unique study design that allowed us to analyze the effects of fasting both immediately after fasting and after refeeding.

## 2 Material and Methods

### 2.1 Animals and Randomization

This study included 30 female C57BL/6J mice that were bred in pathogen‐free (SPF) cages at the Biomedical Research Facility, Vienna, Austria and were transferred to the Medical University of Graz, Austria at 8 weeks of age. After transfer, the animals were housed in individually ventilated cages (EM500, Tecniplast, Hohenpeißenberg, Germany) and allowed to acclimatize for 3 weeks. At 11 weeks of age, ten mice each were randomly assigned to one of three groups: fasting group, refeeding group or control group and rehoused in groups of two mice per cage. The online randomization tool Sealed Envelope was used for randomization [27]. Animals of the fasting and the refeeding group were housed separately because intraperitoneal glucose tolerance tests (IGTT) and sacrifices were performed on different days in these groups. Other than that, the animals of the fasting and the refeeding group received the same treatment.

All animal experiments have been approved by the Austrian government before any study‐related activity was performed. The study was conducted in accordance with Austrian laws and legislations for animal experiments (Tierversuchsgesetz 2012; reference number: BMWFW‐66.010/0160‐WF/V/3b/2017).

### 2.2 Study Design

**FIGURE 1:**
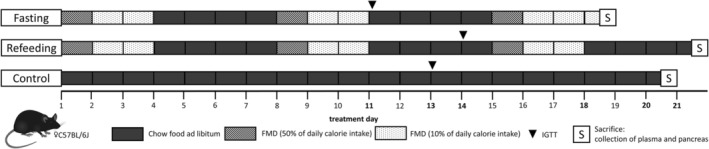
Study design: Thirty female C57BL/6 mice were randomized to fasting, refeeding and control group (10 mice per group). Arrowheads mark the time points of IGTTs and the S at the end of each bar marks the time points of sacrifices and collection of whole blood and pancreas.

Starting at day 1, three weekly fasting/refeeding cycles with 3 days of FMD treatment and 4 days of chow food ad libitum (except for the pre‐IGTT food withdrawal and the duration of the IGTT) at each cycle were administered to both the fasting and refeeding group. Between day 11 and day 14, all mice underwent an IGTT (fasting group: day 11, refeeding group: day 14, control group: day 13, Figure 1). Body weight, water, and food intake were assessed daily, 5 days before the first fasting/refeeding cycle started (at approximately 12 weeks of age). Blood glucose was measured before and after each fasting/refeeding cycle using an Accu‐Check Guide test strip system (Roche, Rotkreuz, Switzerland). The light/dark cycle was set to 12 h/12 h during the entire study.

At the end of the study, animals of all groups were humanely sacrificed with Ketamine/Xylazine (100 mg/kg Ketasol (Livisto, Senden, Germany); 16 mg/kg Rompun (Bayer, Leverkusen, Germany)) [28] 7 days after their IGTTs. Then, whole blood and pancreata were collected and mice decapitated (fasting group: day 18, refeeding group: day 21 and control group: day 20).

### 2.3 Diet and Feeding Schedule

FMD was procured from L‐Nutra Inc. (L‐Nutra Italia S.r.l., Genova, Italy) and prepared as previously described [23]. A total of 44% carbohydrates, 9% protein and 47% fat accounted for a calorie density of 2.92 kcal/g FMD. Normal food intake was calculated based on the average daily food consumption 5 days before the FMD was initially administered.

FMD was supplied on three consecutive days per week. On the first day 50% of the average caloric intake per day (approximately 13.8 kcal) and on the second to third day 10% of the average caloric intake per day were applied, followed by 4 days of access to chow food ad libitum. Between 8:00 and 9:00 a.m. each animal received 5 g HydroGel (ClearH_2_O, SSNIFF Spezialdiäten, Soest, Germany) in addition to the FMD to prevent dehydration and intestinal stress during fasting. Animals of the control group had access to regular chow food (3.225 kcal/g, 67% carbohydrates, 24% protein, 9% fat) ad libitum throughout the study except for the pre‐IGTT food withdrawal and the duration of the IGTT, and they were housed exclusively with animals from their group. During refeeding, animals from the fasting and refeeding group received the same regular chow food as the control group. Access to water was provided ad libitum throughout the study.

### 2.4 Plasma Hormones

Whole blood was collected from right cardiac ventricles of anesthetized mice and transferred to EDTA‐coated tubes at the time of sacrifice. The tubes were immediately centrifuged at 7000 RPM at 4°C for 15 min and the supernatant plasma was transferred into DNA LoBind tubes. Plasma samples were immediately frozen at −196°C.

Plasma concentrations of proinsulin (ALPCO, Salem, USA), insulin (Crystal Chem, Zaandam, the Netherlands), glucagon (Crystal Chem, Zaandam, the Netherlands), leptin (Crystal Chem, Zaandam, the Netherlands), and IGF‐1 (Crystal Chem, Zaandam, the Netherlands) were assessed using the respective ELISA manufacturer protocols (Mouse Proinsulin ELISA, May 29, 2018; Ultra Sensitive Mouse Insulin ELISA Kit v.10/Mar./2019; Mouse glucagon ELISA Kit instruction; Mouse leptin ELISA v.9/Jul/2018; Mouse IGF‐1 ELISA v.4, respectively). Absorption was measured at room temperature using a Spectrostar Omega (BMG LABTECH, Ortenberg, Germany) with predefined absorbance values for each hormone (proinsulin: 450 nm; insulin, glucagon, leptin: 450 and 630 nm).

### 2.5 Intraperitoneal Glucose Tolerance Test (IGTT)

After a 6 h overnight food withdrawal, body weight was measured for dose calculation and mice were single‐housed throughout the duration of the IGTT. At 6:30 a.m. the blood glucose concentration was measured (Accu‐Check Guide test strip system, Roche, Rotkreuz, Switzerland) and 1.5 mg intraperitoneal glucose (sterilely filtered D‐glucose in phosphate‐buffered saline) per g body weight was injected. Blood glucose concentration was measured at 15, 40, 60, and 120 min in the fasting group, and at 15, 30, 60, and 120 min in the refeeding and the control group. No food was supplied during the IGTT. After 120 min, the mice were transferred back to their home cages and further treated according to the protocol.

### 2.6 Immunohistochemistry Procedures

Directly after extraction, the pancreata were divided into four parts: Head, body, medial tail, and lateral tail. The lateral tail was immediately minced and immersed in 0.1 M cacodylate buffer (pH 7.4, 2.5% glutaraldehyde and 2% paraformaldehyde).

Head, body and medial tail of the pancreata were fixed in 10% neutral buffered formalin (H0897/1255, Süsse Labortechnik, Gudensberg, Germany) at 4°C for at least 24 h. Using a Tissue‐Tek VIP machine (Sakura Finetek Austria, Vienna, Austria), the formalin‐fixed tissues were once more fixed with formalin (6%, 40°C, 1 h, two times), dehydrated in an ascending ethanol series (70% Ethanol, 40°C, 1 h; 80% Ethanol, 40°C, 1 h; 96% Ethanol, 40°C, 1 h, two times; 100% Ethanol, 40°C, 1 h, two times), optically cleared and immersed in paraffin. Then, the processed pancreatic tissues were immersed in Histolab clear (#14250, Histolab, Askim, Sweden) at 40°C for 1 h (two times), in paraffin at 53°C for 1 h (two times) and again in paraffin at 53°C for 2 h (two times). Finally, the pancreatic tissues were automatically embedded in paraffin blocks (56°C, ATS‐200856, Sanova, Vienna, Austria) using a Tissue‐Tek TEC machine (Sakura Finetek Austria, Vienna, Austria). Ten sections, each 2 μm thick, were cut from the paraffin blocks containing the embedded pancreatic tissue using a Microm HM355S (Thermo Fisher Scientific, Vienna, Austria). These sections were then mounted on Epredia SuperFrost Plus Adhesion slides (J1800AMNZ, Thermo Fisher Scientific, Vienna, Austria) and dried at 37°C overnight. The innermost sections were used for fluorescent multiplexed immunohistochemistry (fm‐IHC) staining.

Fm‐IHC staining was performed with a BOND RX Fully Automated Research Stainer (Leica Biosystems, Vienna, Austria) and reagents from the Opal 7‐Color Automation IHC Kit, including counterstaining with Spectral 4′,6‐Diamidin‐2‐phenylindol (DAPI) (NEL821001KT, Akoya Biosciences, Marlborough, Massachusetts, USA). Insulin (1:60000, ab181547, Abcam, Cambridge, UK), glucagon (1:5000, ab92517, Abcam, Cambridge, UK), somatostatin (1:12000, ab111912, Abcam, Cambridge, UK), PDX1 (1:400, #5679, Cell Signaling Technology, Danvers, Massachusetts, USA), and BRN4 (1:100, NBP1‐89934, Novus Biologicals, Thermo Fisher Scientific, Vienna, Austria) were used as primary antibodies. Dako EnVision+ System‐HRP 2nd anti‐rabbit (Agilent Technologies, Vienna, Austria) was used as the secondary antibody.

The stained pancreatic tissue sections were then manually mounted with a coverslip using ProLong Diamond Antifade Mountant (P36970, Thermo Fisher Scientific, Vienna, Austria). All slides were digitized with the microscope scanning platform Vectra3 and Vectra Polaris (Akoya Biosciences, Marlborough, Massachusetts, USA). Whole‐slide multispectral images were acquired with at least 20‐fold magnification. Spectral unmixing was performed in Phenochart using the respective internal libraries (Akoya Biosciences, Marlborough, Massachusetts, USA). Digital multispectral images were imported into the Halo software (Indica Labs, Albuquerque, New Mexico, USA), fused and saved as whole‐slide multispectral images. The High‐Plex FL v2.0 module in Halo was used to segment cells based on DAPI signals and to detect marker‐positive cells by setting individual thresholds for each section. Islet positivity was defined by a minimum of three positive cells for insulin or glucagon. Blinded researchers set the thresholds and annotated the islets manually based on histological standards. β‐cells were defined as insulin^+^glucagon^−^ and α‐cells were defined as insulin^−^glucagon^+^. Marker‐positive cells were quantified and expressed as the proportion of marker‐positive cells relative to the total number of DAPI‐positive nuclei within each islet.

### 2.7 Electron Microscopy Procedures

Pancreatic tissue buffered in 0.1 M cacodylate was fixed in 1% osmium tetroxide at room temperature for 2 h and was then transferred into 50%, 70%, 90%, and 100% ethanol. Each step lasted 10 to 20 min. Next, pancreatic tissues were infiltrated with ethanol and pure epoxy resin (TAAB, Aldermaston, UK) before being placed in embedding molds with pure epoxy resin (TAAB, Aldermaston, UK) for 8 h. Afterwards, the pancreatic tissue blocks were polymerized at 60°C for 48 h, and sections of the pancreatic tissue blocks were cut at 70 nm using a UC 7 Ultramicrotome (Leica Microsystems, Vienna, Austria). Then these sections were stained with lead citrate and platin blue for 5 min/15 min, respectively. A FEI Tecnai G2 20 transmission electron microscope (Thermo Fisher Scientific, Vienna, Austria) and a Gatan ultrascan 1000 charge coupled device (CCD) camera (−20°C, Gatan, Munich, Germany) using the acquisition software Digital Micrograph (Gatan, Munich, Germany) were used to acquire TEM images (Serial EM) at an acceleration voltage of 120 kV.

Scanning transmission electron microscopy (STEM) imaging mode of a field emission scanning electron microscope (ZEISS FE‐SEM Sigma 500, Zeiss, Germany) with an acceleration voltage of 15 kV in combination with ATLAS TM (Zeiss, Germany) was used to correlate TEM and STEM images on large areas of pancreatic tissue with high‐resolution AZoNano (2021) (AzoNetwork, UK). This led to whole islet sections and improved target localization of the imaged areas. Single whole islet sections of similar size (maximum one per mouse; control group *n* = 4, fasting group *n* = 3) were selected.

On an ultrastructural level, β‐cell granules typically appear as granules with a diameter of about 350 nm with halos, unlike α‐cells, which often appear with thin halos, and δ‐cell granules, which appear without halos [29, 30]. Mature granules were defined as granules with clear distinction between an electron‐beam dense core and a highly penetrable halo [30]. Immature granules were defined as granules with a large core with indistinct separation from a narrow halo and not specifically annotated [29].

A DeepLabV3 network with a ResNet‐50 backbone [31] was fine‐tuned for this study by replacing the final convolutional layer with four output channels (background, α‐, mature β‐ and δ‐cell granules) to generate segmentation masks for STEM images. This segmentation network was trained using the Adam optimizer with a learning rate of 1 × 10^−4^ [32] on 23 pixel‐wise annotated TEM images of pancreatic tissue sections. The TEM image sizes were then digitally adapted to align with those of the STEM images. Subsequently, each generated STEM image was split into overlapping tiles (each 2024 × 1024 pixels), and these tiles were used as an input to the trained segmentation network to generate semantic masks for the cell granule classes *α*, *β*, and *δ*. Subsequently, the obtained segmentation masks were reassembled into full semantic masks covering the entire STEM acquisition. We applied a watershed algorithm [33] to those full semantic masks to separate the individual granule classes, thus yielding a final mask in which each detected granule was labeled *α*, *β*, or *δ*. From these final masks, we extracted per‐class counts, mean granule areas, and core‐to‐halo ratios.

### 2.8 Statistical Analyses

Statistical analyses were carried out using SPSS version 26 or Graphpad Prism 9.5.1 for Windows. The area under the blood glucose concentration versus time curve (AUC) during the IGTT was computed with Graphpad Prism 9.5.1 for Windows only. Shapiro‐Wilk and F‐test were performed to assess normality and variance of samples. Results are displayed as mean ± SD unless stated otherwise. Mean values between FMD and control group were compared using *t*‐test when data were normally distributed, and Mann‐Whitney *U*‐test when data were non‐normally distributed. Analysis of variance (ANOVA, for normally distributed data), Welch's ANOVA (for data with inhomogeneous variances) or Kruskal‐Wallis tests (for non‐normally distributed data) were used to compare the values of the individual groups. Multiple comparisons were corrected by Bonferroni (for data with homogenous variances), Tamhane T2 (for data with inhomogeneous variances) or Dunn's post hoc tests (for non‐normally distributed data). A *p* value ≤ 0.05 was considered statistically significant.

One mouse from the fasting group was excluded from the analysis after sacrifice on day 18 due to its initial and overall lower body weight. Nevertheless, this mouse stayed included in the study until it was sacrificed to prevent bias in the data that could have arisen from different numbers of mice per cage. No statistical comparison of the fasting/refeeding group to the control group was performed at day 4 because no blood glucose samples from seven mice from the fasting/refeeding group could be measured due to limitations of getting a sufficient blood sample volume. One glucagon concentration value of the control group was below the lower detection limit and was therefore excluded from further data analyses.

Further, the volume of a plasma sample of one mouse from the fasting group was below the required limit of detection to measure IGF‐1 plasma concentration and the concentration value of this sample was thus not available for analysis.

Two samples were excluded from the fm‐IHC analysis after staining. One of which, a sample from the refeeding group, was excluded due to a technical failure to scan the stained slide, and the other one, a sample from the control group, had too poor formalin fixed paraffin‐embedded quality to be further analyzed.

## 3 Results

### 3.1 Fasting Cycles Temporarily Lowered Bodyweight and Blood Glucose Concentrations Under the Same Cumulative Food Intake

**FIGURE 2:**
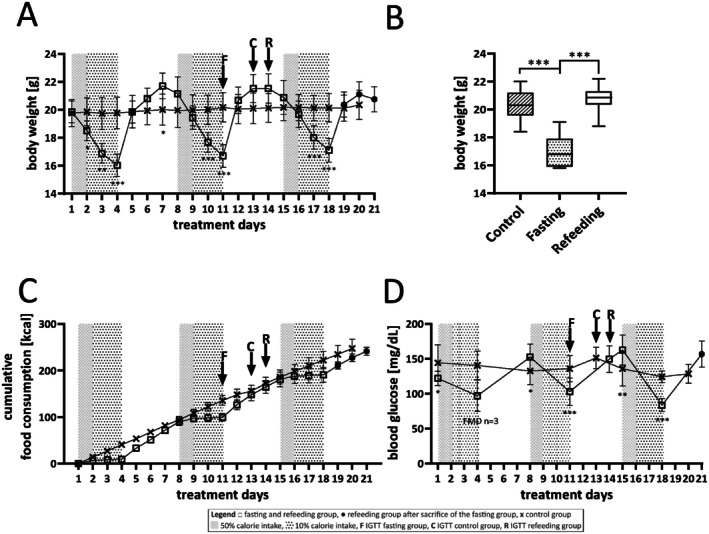
Body weight as a function of treatment days (A), body weight at sacrifice (B), cumulative food consumption (C), and blood glucose (D) as a function of treatment days of the fasting (*n* = 9), refeeding (*n* = 10), and control group (*n* = 10). □: Fasting and refeeding group (*n* = 19); ●: Refeeding group after sacrifice of the fasting group (*n* = 10); x: Control group (*n* = 10). Checkerboards (50% calorie intake) and small dots (10% calorie intake) indicate fasting cycles. Arrows indicate IGTTs for the fasting group (F, day 11), the control group (C, day 13), and the refeeding group (R, day 14). Data are presented as mean ± SD. *p < 0.05, **p < 0.01, ***p < 0.001.

Overall, the FMD treatment was well tolerated. The body weights in the control group were almost stable over the entire course of the study, whereas the body weights in the fasting/refeeding group showed fluctuations corresponding to the respective caloric intake during the fasting and refeeding cycles. Markedly, significant differences between body weights of the fasting/refeeding and the control group were already evident in each fasting cycle after 2 days of fasting (day 3: −14.4% ± 3.5%, day 10: −11.7% ± 3.5%, day 17: −10.5% ± 4.0%; *p* < 0.001 each). However, after 1 day of refeeding in each cycle (days 5, 12, 19), the body weights of mice of the fasting/refeeding group matched or tended to exceed those of the control group again (Figure 2A). The body weights at the time point of sacrifice were significantly higher in mice of the control and the refeeding group compared to those of mice of the fasting group (*p* < 0.001, Figure 2B). The cumulative food consumption over three fasting/refeeding cycles was similar in the fasting and the refeeding group, but its course differed between the fasting/refeeding and the control group (Figure 2C). The cumulative food consumption over time of mice from the fasting and the refeeding group did not exceed that of the control group at any point in time throughout the study (Figure 2C).

Blood glucose concentrations at study start (day 1) were significantly higher in the control group than in the fasting and refeeding group. They were stable throughout the study duration in the control group, showed decreasing trends during the fasting cycles, and increasing trends during the refeeding cycles in the fasting/refeeding group (Figure 2D). Blood glucose concentrations were significantly decreased after the second and third fasting cycle in the fasting/refeeding group compared to the control group (day 11, day 18, *p* < 0.001, respectively). Conversely, blood glucose concentrations were significantly increased after the first and second refeeding cycle in the fasting/refeeding group compared to the control group (day 8, *p* < 0.05, day 15, *p* < 0.01) (Figure 2D).

### 3.2 Fasting/Refeeding Cycles Increased the Number of Insulin^+^Glucagon^+^ Cells and Expression of BRN4

After three fasting/refeeding cycles the number of cells that stained positive for both insulin and glucagon (insulin^+^glucagon^+^ cells), normalized to the total number of DAPI‐positive nuclei within each islet, increased in the fasting group (*p* = 0.03) and showed a tendency to be increased in the refeeding group compared to the control group (Table 1).

**TABLE 1:** Endocrine cells per islet in the fasting, refeeding and the control group shown as marker‐positive cells normalized to the total number of DAPI‐positive cell nuclei within each islet from fm‐IHC of pancreatic tissue. Unless otherwise stated, profiles can be positive or negative for any other marker, e.g., insulin‐positive cells include all cells that stained positive for insulin, regardless of any other markers.

Endocrine lineage	Staining profile	Controls (mean ± SD)	Fasting (mean ± SD)	*p* (vs. Controls)	Refeeding (mean ± SD)	*p* (vs. Controls)
Insulin^+^	Insulin^+^	0.53 ± 0.24	0.55 ± 0.22	NS	0.56 ± 0.22	NS
	β‐cells (Insulin^+^Glucagon^−^)	0.38 ± 0.28	0.35 ± 0.27	NS	0.4 ± 0.27	NS
	Insulin^+^PDX1^+^Glucagon^−^BRN4^−^	0.36 ± 0.27	0.32 ± 0.25	NS	0.34 ± 0.26	NS
	Insulin^+^Glucagon^+^	0.15 ± 0.19	0.20 ± 0.24	0.03	0.16 ± 0.20	NS
Glucagon^+^	Glucagon^+^	0.36 ± 0.29	0.37 ± 0.31	NS	0.34 ± 0.30	NS
	α‐cells (Insulin^−^Glucagon^+^)	0.21 ± 0.19	0.17 ± 0.18	NS	0.19 ± 0.19	NS
	Insulin^−^PDX1^−^Glucagon^+^BRN4^+^	0.02 ± 0.04	0.03 ± 0.06	< 0.05	0.02 ± 0.07	NS
Others	PDX1^+^	0.6 ± 0.22	0.58 ± 0.21	NS	0.54 ± 0.24	< 0.01
	BRN4^+^	0.04 ± 0.07	0.08 ± 0.15	< 0.001	0.05 ± 0.09	NS
	PDX1^+^BRN4^+^	0.01 ± 0.04	0.03 ± 0.11	< 0.05	0.01 ± 0.03	NS
	Somatostatin^+^	0.05 ± 0.08	0.06 ± 0.09	NS	0.05 ± 0.08	NS

The number of any cells staining positive for insulin (insulin^+^ cells) and glucagon (glucagon^+^ cells), normalized to the total number of DAPI‐positive nuclei within each islet, were comparable among the fasting, refeeding, and the control group. Similarly, the normalized number of β‐cells (insulin^+^glucagon^−^) and α‐cells (insulin^−^glucagon^+^) did not differ between the three groups. Notably, the number of cells staining positive for glucagon and BRN4 (insulin^−^PDX1^−^glucagon^+^BRN4^+^cells), normalized to the total number of DAPI‐positive nuclei within each islet, was significantly increased in the fasting group (*p* < 0.05).

The number of any cells staining positive for BRN4 (BRN4^+^ cells), normalized to the total number of DAPI‐positive nuclei within each islet, was significantly higher in the fasting group (*p* < 0.001) and showed an increasing trend in the refeeding group compared to the control group. In accordance with these findings, we found a threefold increase of cells staining positive for both PDX1 and BRN4 (PDX1^+^BRN4^+^ cells), normalized to the total number of DAPI‐positive nuclei within each islet, in the fasting compared to the control group (*p* < 0.05). In the refeeding group, the number of PDX1^+^BRN4^+^ cells normalized to the total number of DAPI‐positive nuclei within each islet was comparable to that of the control group.

The overall number of islet cells and the overall endocrine area (μm^2^ × 10^3^ ± SD) per animal showed a tendency to be increased after fasting (49.3 ± 19.6; 361 ± 208) and refeeding (43.9 ± 15.0; 338 ± 230) compared to the control group (34.1 ± 21.3; 206 ± 155).

### 3.3 Fasting Increased β‐Cell Granule Core‐To‐Halo Ratio

**FIGURE 3:**
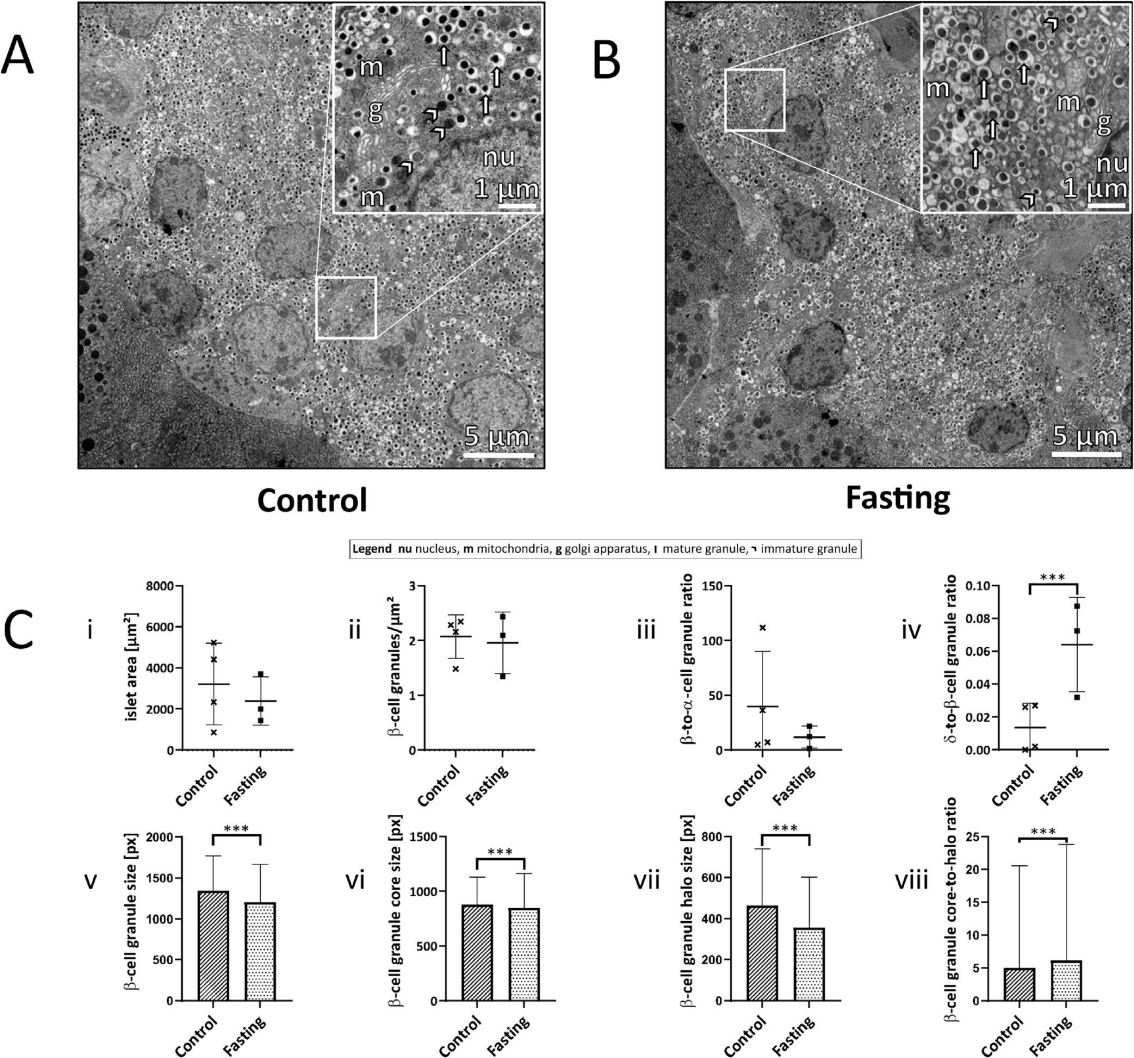
Ultrastructure of pancreatic islets. (A, B) Selected STEM images of whole‐islet sections of the control group (A) and the fasting group (B). Enlarged sections of a β‐cell (nu = nucleus, m = mitochondria, g = golgi apparatus, arrow = mature granule, arrowhead = immature granule). (C) Islet area (i), β‐cell granules/μm^2^ (ii), β‐to‐α cell granule ratio (iii), δ‐to‐β‐cell granule ratio (iv), β‐cell granule size (v), β‐cell granule core size (vi), β‐cell granule halo size (vii), and β‐cell granule core‐to‐halo ratio (viii) of the control and the fasting group. Control group (hatched, *n* = 4); fasting group (dotted, *n* = 3). One pixel (px) corresponds to 4 nm. Data are presented as mean ± SD. ***p < 0.001.

On an ultrastructural level, pancreatic tissue sections from the fasting group exhibited widened endoplasmic reticulum (ER) compared to the control group and aggregated mitochondria (Figure 3A,B). Additionally, in contrast to the control group, pancreatic tissue sections from the refeeding group showed disordered α‐ and δ‐cell granule areas and active mitochondria (Figure S1).

The whole islet areas, the number of β‐cell granules/μm^2^, and the β‐to‐α‐cell granule ratios were comparable in the fasting and the control group (Figure 3C, i – iii). In addition, the numbers of α‐cell granules and δ‐cell granules were similar in the fasting and control group (Figure S2). However, we observed a significant increase in the δ‐to‐β‐cell granule ratio in the fasting compared to the control group (*p* < 0.001, Figure 3C, iv).

Furthermore, the size of the β‐cell granules was significantly decreased in the fasting compared to the control group (*p* < 0.001, Figure 3C, v). The sizes of the β‐cell granule core and of the β‐cell granule halo were significantly decreased in the fasting group compared to the control group (*p* < 0.001 each, Figure 3C, vi, vii). However, the core‐to‐halo ratio of β‐cell granules was significantly higher in the fasting than in the control group (*p* < 0.001, Figure 3C, viii).

### 3.4 Fasting Cycles Reduced Plasma Proinsulin and IGF‐1, but Not Insulin Concentrations

**FIGURE 4:**
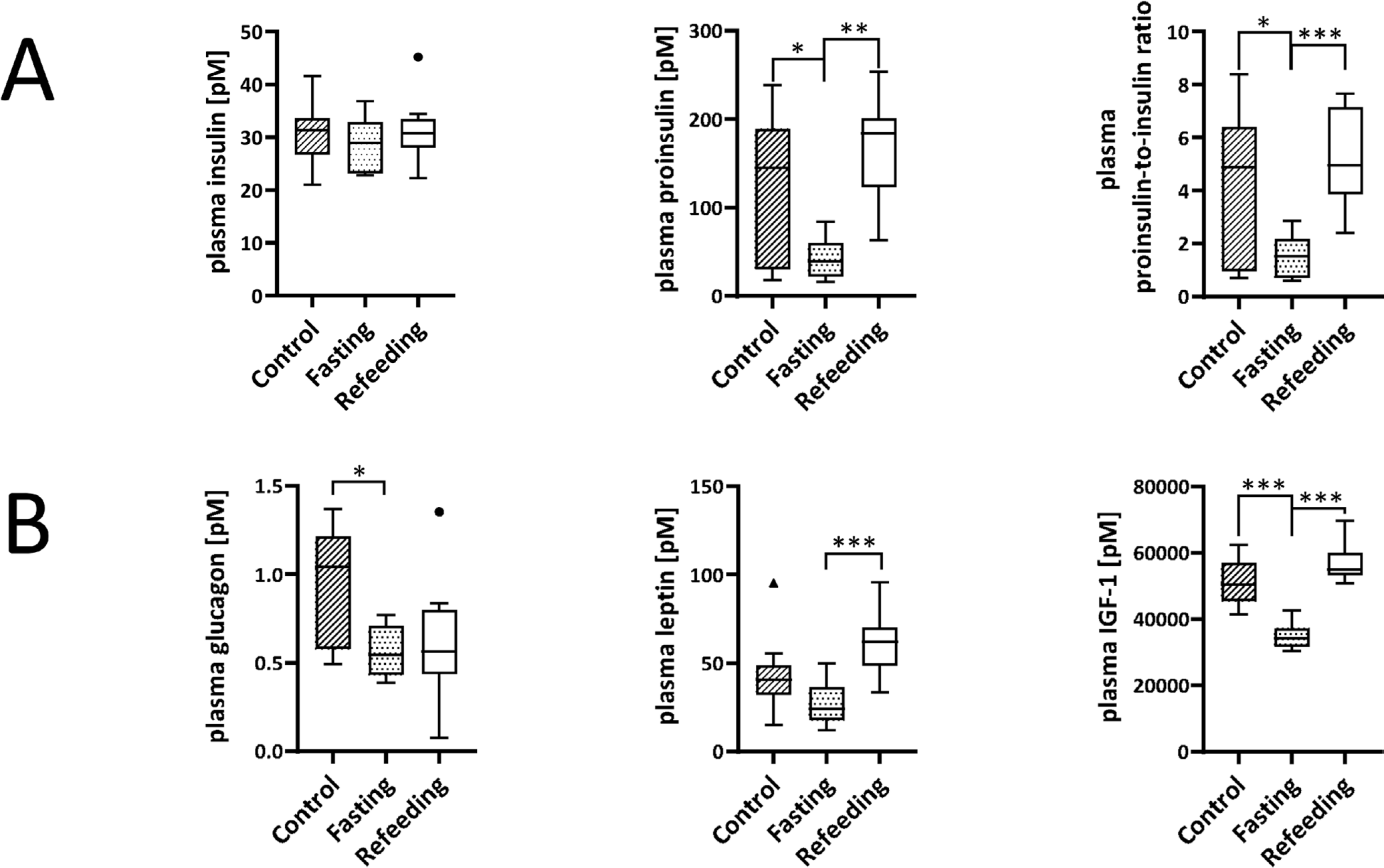
Plasma hormone concentrations at the time point of sacrifice of the fasting (dotted, *n* = 9), refeeding (white, *n* = 10) and control group (hatched, *n* = 10). (A) Plasma insulin (left side), proinsulin (middle) and proinsulin‐to‐insulin ratio (right side). (B) Plasma glucagon (left side), leptin (middle), and IGF‐1 (right side). Data are presented as mean ± SD. *p < 0.05, ***p < 0.001.

Plasma insulin concentrations were comparable in all three groups at the time point of sacrifice of the respective group (Figure 4A, left side). In contrast, the plasma proinsulin concentrations were significantly decreased in the fasting compared to the refeeding (*p* < 0.001) and the control group (*p* < 0.01, Figure 4A, middle). Consequently, the plasma proinsulin‐to‐insulin ratio was significantly lower in the fasting compared to the refeeding (*p* < 0.001) and the control group (*p* < 0.05, Figure 4A, right side).

Plasma glucagon concentrations were significantly decreased in the fasting group and showed a tendency to be decreased in the refeeding group compared to the control group at the time point of sacrifice of the respective group (Figure 4B, left side). Plasma concentrations of leptin were significantly increased in the refeeding group compared to the fasting group (*p* < 0.001, Figure 4B, middle). The plasma concentrations of IGF‐1 were significantly decreased in the fasting group (*p* < 0.001) compared to the refeeding and the control group (Figure 4B, right side).

### 3.5 Fasting Cycles Temporarily Impaired Glucose Tolerance

**FIGURE 5:**
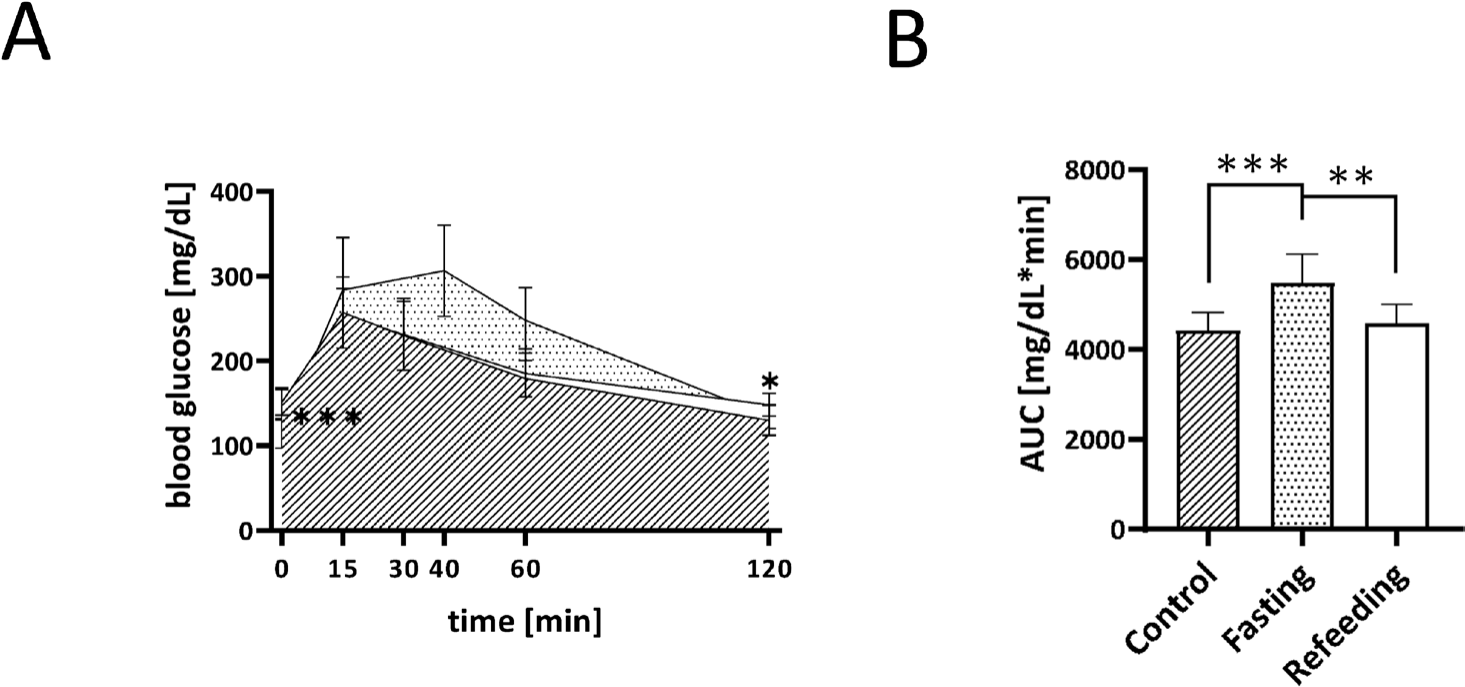
Blood glucose concentrations as a function of IGTT duration from 0 to 120 min (A) and respective AUCs (B) of the fasting (dotted, *n* = 9), refeeding (white, *n* = 10), and control group (hatched, *n* = 10). The fasting group received the IGTT at day 11 (after fasting), the refeeding group at day 14 (after 3 days of refeeding) and the control group at day 13. Data are presented as mean ± SD. ***p < 0.001, **p < 0.01, *p < 0.05.

Based on significantly lower blood glucose concentrations in the fasting group than in the refeeding and the control group at the start of the IGTT (0 min, *p* < 0.001), blood glucose concentrations were higher in the fasting than in the refeeding and the control group after approximately 15 min, continued to increase to a peak at 40 min and tended to remain higher (Figure 5A). The blood glucose concentration in the control group peaked at around 15 min (Figure 5A). Even though blood glucose concentrations of the refeeding and control group were not significantly different at the beginning of the IGTT, they were significantly increased in the refeeding but not in the fasting group compared to the control group at 120 min (*p* < 0.05). AUC of the fasting group was significantly higher compared to those of the refeeding (*p* < 0.01) and the control group (*p* < 0.001) (Figure 5B).

## 4 Discussion

Immunohistochemical, ultrastructural and metabolic analyses revealed that three intermittent FMD cycles led to increased plasticity of pancreatic islet cells, as indicated by increased numbers of cells staining positive for both insulin and glucagon insulin^+^glucagon^+^ cells and both PDX1 and BRN4 (PDX1^+^BRN4^+^cells). In addition, the architecture of pancreatic β‐cell granules was markedly altered and we observed a reduction in proinsulin and IGF‐1 concentrations while insulin concentrations remained unchanged after fasting. By employing a unique study design, specifically the separate assessment of fasting and refeeding, we were able to distinguish acute effects, such as changing insulin sensitivity and glucose tolerance, from more sustained effects on β‐cells and islet numbers.

Our findings expand on systemic observations from previous studies [17, 22, 23, 26] by addressing temporal aspects of pancreatic islet cell plasticity, which have not been covered in detail previously.

Even though the number of any pancreatic islet cells staining positive for insulin or for glucagon did not change, the number of any pancreatic islet cells staining positive for both insulin and glucagon (insulin^+^glucagon^+^ cells) significantly increased in the fasting group and showed a tendency to be increased in the refeeding group compared to the control group. It has been assumed that insulin^+^glucagon^+^ cells may serve to protect cells from cytokine‐induced apoptosis [34], may indicate post‐developmental endocrine cell transdifferentiation [35, 36] or are associated with impaired glucose tolerance after fasting [37].

In our study we analyzed fasting and refeeding separately and observed an increase in the number of insulin^+^glucagon^+^ cells in both groups. This finding suggests that this effect is more related to the regeneration of islet cells rather than to insulin resistance. Furthermore, we observed that the number of β‐cells and α‐cells remained unchanged after fasting and after refeeding. This is in contrast to previously observed increased numbers of α‐cells and β‐cells together with the endocrine cell precursor marker Ngn3^+^ after FMD treatment [23, 26].

We further found increased numbers of cells staining positive for PDX1 and BRN4 (PDX1^+^BRN4^+^ cells) as well as increased numbers of any cells staining positive for either BRN4 after fasting, and to a lesser extent also after refeeding. Therefore, we propose that BRN4, an important transcription factor for the transition from endocrine progenitor cells to α‐cells during the development of the pancreas [38, 39, 40], could serve as a new, specific α−/β‐cell regeneration marker after fasting/refeeding. Although immunostaining of pancreatic polypeptide cells and epithelial cells with BRN4 has been described previously, insulin‐positive or somatostatin‐positive cells generally do not express BRN4 [40]. Interestingly, pancreatic islet cells from BRN4 knockout mice have been shown to lack the transcription factor PDX1, even though BRN4 and PDX1 are typically not co‐expressed in adult mice [40]. It is therefore not surprising that in our study, a subset of cells staining positive for glucagon and BRN4, but negative for insulin and PDX1 (insulin^−^PDX1^−^glucagon^+^BRN4^+^ cells), was also increased after fasting.

Taking all our immunohistochemical findings together, we suggest that fasting triggers substantial islet regeneration, underlining a far larger islet plasticity than has been generally anticipated.

While reduction of IGF‐1 concentrations has been reported after prolonged fasting and FMD before [17, 23, 24], activation of somatostatin receptors has also been reported to decrease IGF‐1 concentrations [41]. Low concentrations of IGF‐1 have also been reported to protect against cellular stress [42] or lead to multi‐system regeneration [17].

In addition to immunohistochemical analyses in our study, we also performed in‐depth electron microscopy imaging. Interestingly, the δ‐to‐β‐cell granule ratio was three‐fold higher after fasting than after refeeding. Fasting led to a significantly decreased size of mature β‐cell granules but not to fewer β‐cell granules per islet area in our study. This is in line with results from a previous study showing that insulin secretion from β‐cell granules depends on the current glucose concentrations rather than on the granule size [43]. We further observed significant differences in β‐cell granule core and halo sizes. Although the β‐cell granule halo sizes also decreased significantly, the magnitude of this decrease was smaller than that of the β‐cell granule cores. Consequently, the core‐to‐halo ratio in mature β‐cell granules was increased, indicating a higher degree of crystallization and, therefore, enhanced insulin secretory capacity following fasting.

We found, next to decreased glucagon concentrations, consistent insulin concentrations in plasma after fasting and refeeding, which contrasts with results from previous studies [17, 22, 23, 26]. One possible explanation for this discrepancy might be the differences in study design, e.g., the different time points of sampling. Whereas we collected the plasma samples at the third fasting day during the third fasting/refeeding cycle (day 18), in these previously performed studies plasma samples have been collected after 4 to 5 days of fasting [17, 22] or several days after the last fasting/refeeding cycle [23]. Another reason for the diverging results could be the different insulin responses of animals with different metabolic conditions: While previous studies have investigated male mice [22] or a T2D murine model [26], our study investigated healthy female mice.

We further observed a reduction of proinsulin while insulin plasma concentrations remained constant after fasting. This underlines the notion that a reduced proinsulin‐to‐insulin ratio can be a sensitive marker for β‐cell function [44], and an indicator for protection from stress in the ER of β‐cells [45]. Hence, our results indicate that FMD treatment not only increases islet plasticity after fasting and refeeding but also protects β‐cells against stress.

Notably, we employed a unique study design in which insulin resistance was assessed immediately after fasting and after refeeding separately and we found that glucose tolerance was transiently impaired after fasting and normalized upon refeeding. This was in contrast to results from previous studies where improved glucose tolerance has been observed only after refeeding by assessing the Homeostatic Model Assessment Indices HOMA‐B and HOMA‐IR, or IGTT following a minimum of four cycles of FMD in a murine model of T2D [23, 26], or HOMA‐IR in healthy individuals after completion of the third FMD cycle [21]. Mechanistically, we observed a substantial increase in hepatic lipid content (data not shown), which is in line with previous findings [22, 46, 47].

One limitation of this study is the low sample size, which leads to limited statistical significance in some immunohistochemical parameters. However, the limited number of animals was in accordance with the ethical principles of animal experimentation, i.e., Reduce, Replace, Refine [48].

Another limitation is that we investigated the effects of FMD in healthy mice and not yet in a diabetic mouse model. Nevertheless, our findings provide novel insights into islet plasticity far beyond current knowledge.

One strength of our study is that we were analyzing the effects of fasting and refeeding separately. This unique study design allowed us to distinguish between acute effects, such as changing insulin sensitivity and glucose tolerance, and sustained effects on β‐cells and islet numbers after fasting and refeeding.

Other strengths of our study include the use of advanced multi‐channel immunohistochemical methods allowing for co‐staining of pancreatic islet cell markers and thus highlighting new aspects of islet plasticity. Also, the use of machine learning enabled the analysis of thousands of ultrastructural β‐cell granules, thereby reducing inter‐ and intra‐observer variabilities.

## 5 Conclusion

Three intermittent FMD cycles increased plasticity in pancreatic islet cells, by increased numbers of cells staining positive for both insulin and glucagon, and cells staining positive for both PDX1 and BRN4. Also, the ultrastructure of pancreatic β‐cell granules was markedly changed with β‐cells exhibiting a higher proportion of crystallized mature granules and, consequently, an increased insulin secretory capacity following fasting. The observed reduction of proinsulin‐to‐insulin ratio after fasting suggests improved β‐cell function and reduced ER stress in pancreatic ß‐cells.

Our findings indicate that prolonged intermittent FMD treatment may offer a therapeutic window that can promote β‐cell regeneration in both T1D and T2D. We propose that the observed plasticity of pancreatic islet cells could provide a basis for new concepts for β‐cell regeneration in vivo.

However, additional studies are required to determine the optimal frequency and duration of FMD cycles necessary to induce β‐cell regeneration, as well as to elucidate the underlying molecular mechanisms and their effects in animal models of T1D and β‐cell‐deficient T2D.

## Author Contributions

Conceptualization: C.M.H., P.K., T.R.P.; Investigation: C.M.H., B.E., L.B., K.B., D.P., J.F., D.K.; Methodology: C.M.H., B.E., L.B., K.B., D.P., J.F., P.K., D.K., T.R.P.; Resources: J.K., A.P., B.P., D.K., T.R.P.; Software: B.E., L.B.; Formal analysis: C.M.H., L.H.; Visualization: C.M.H, B.B., D.P., D.K., T.R.P.; Writing – original draft preparation: C.M.H., B.B.; All authors reviewed and revised the manuscript for important intellectual content and gave final approval of the manuscript to be published. T.R.P. is the guarantor of this work.

## Funding

This work was supported by BioTechMed‐Graz (BIOTECHMEDGRAZ), MIDAS. Breakthrough T1D (JDF), COMET – Common Mechanisms in Autoimmune Diseases. Austrian Science Fund (FWF), 10.55776/P34109,10.55776/P29328,10.55776/I3165, 10.55776/P29328,10.55776/PAT1744223.

## Conflicts of Interest

The authors declare no conflicts of interest.

## Supporting information


**Figure S1:** Representative transmission electron microscopy sections from the control (left), fasting (middle), and refeeding (right) group.
**Figure S2:** α‐cell granules (A) and δ‐cell granules (B) from scanning transmission electron microscopy sections of the control and fasting group. Control group (hatched, *n* = 4); fasting group (dotted, *n* = 3). Data are presented as mean ± SD.

## Data Availability

The data that support the findings of this study are available in the Materials and Methods, Results, and/or Figures S1 and S2 of this article.
