# Supplementary material for: Effects of Intermittent Fasting‐Mimicking Diet on Pancreatic Islet Plasticity: Immunohistochemical, Ultrastructural, and Metabolic Profiles
Source: FASEB J. 2026 May 4;40:e71858. doi: 10.1096/fj.202504830RR (PMC13138363; doi:10.1096/fj.202504830RR)
Supplement: Supplementary file 1 — Figure S1: Representative transmission electron microscopy sections from the control (left), fasting (middle), and refeeding (right) group. Figure S2: α‐cell granules (A) and δ‐cell granules (B) from scanning transmission electron microscopy sections of the control and fasting group. Control group (hatched, n = 4); fasting group (dotted, n = 3). Data are presented as mean ± SD. [file FSB2-40-e71858-s001.zip › 202504830RR-sup-0004-SI_Text-S02.docx]

**Supplementary Figure 2**: α-cell granules (A) and δ-cell granules (B) from scanning transmission electron microscopy sections of the control and fasting group. Control group (hatched, n = 4); fasting group (dotted, n = 3). Data are presented as mean ± SD.
